# Prevalence and factors associated with inconsistent condom use among female sex workers in Ethiopia: findings from the national biobehavioral survey, 2020

**DOI:** 10.1186/s12889-023-17253-8

**Published:** 2023-12-04

**Authors:** Muhammed Ahmed Rameto, Saro Abdella, Jemal Ayalew, Masresha Tessema, Jaleta Bulti, Fayiso Bati, Sileshi Lulseged

**Affiliations:** 1 Ethiopian Public Health Institute, Addis Ababa, Ethiopia; 2 Departments of Statistics, College of Natural Science, Wollo University, Dese, Ethiopia; 3 College of Health Sciences, Addis Ababa University, Addis Ababa, Ethiopia

**Keywords:** Female sex worker, Inconsistent condom use, Prevalence, HIV, Ethiopia

## Abstract

**Background:**

The HIV prevalence among Ethiopian female sex workers (FSWs) is estimated to be around 18.5%, which implies that FSWs' sexual partners are significantly exposed to HIV infection and that may be a major factor in HIV transmission in the community. However, it has long been known that using condoms correctly and consistently is an extremely cost-effective global method for preventing HIV infection, but inconsistent condom use (ICU) would pose the greatest proximal risk of HIV acquisition and transmission. Understanding the prevalence and associated risk factors of inconsistence condom use among FSWs would inform policymakers to design programmatic interventions in the context of Ethiopia.

**Methods:**

This analysis used data from the ‘National HIV and STIs Bio-behavioral Survey (NHSBS)’, which was conducted between December 2019 up to May 2020 by using a respondent-driven sampling (RDS) technique among FSWs aged 15 years and older who were selling sex in selected major cities and towns in Ethiopia. A multi-level logistic regression model was fitted to assess town and individual-level variations simultaneously to adjust hierarchical variations. Statistical significance was determined by using a *P*-value less than 0.05 with a 95% confidence interval (CI) not including one.

**Results:**

Overall, 6,085 FSWs from 16 cities and towns participated in the study. The prevalence of inconsistent condom use across the 16 cities and towns was 17.1% [95% CI (16.5, 17.8)]. Inconsistent condom use was significantly higher among FSWs who had depression compared to those without depression [AOR = 1.43; 95% CI (1.13,1.82)], used any drug [AOR = 1.43; 95% CI (1.14–1.79)], had history of sexual violence [AOR = 1.75; 95% CI (1.43, 2.16)], changed sex selling location [AOR = 1.27; 95% CI (1.06, 1.51)], longer period of sex selling experience [AOR = 3.01; 95% CI (2.27, 3.99)], ever had anal sex [AOR = 2.74; 95% CI (2.15, 3.5)], had ≥ 2 non-paying sexual partner [AOR = 2.99; 95% CI(2.26, 3.95)], selling sex `in more than two cities [AOR = 3.01;95% CI (2.27, 3.99)], who lacked access to condom [AOR = 2.1; 95% CI (1.69, 2.67)], and did not have HIV knowledge [AOR = 1.39; 95% CI (1.15, 1.68)].

**Conclusion:**

Inconsistent condom use among FSWs is prevalent in Ethiopia and is associated with marital status, education status, depression, alcohol drinking, drug use, sexual violence, being raped, lack of knowledge about HIV, practising anal sex, selling sex in different locations, having more than two non-paying sexual partners, working in more than two cities, and lack of access to condom at the workplace. Programme interventions to enhance consistent condoms use among FSWs need to take these factors into consideration.

## Introduction

There are approximately 37 million HIV-positive individuals in the world, and the epidemic's challenges have not abated. This is, without a doubt, the worst epidemic in history, resulting in a complex, multifaceted crisis on a massive scale.

Sub-Saharan Africa (SSA), home to over three-quarters (70%) of the world's HIV cases, is where the epidemic has the biggest detrimental effects, with women accounting for a higher percentage of cases (59%) and overall [1]. With an estimated adult HIV prevalence of 0.96% in Ethiopia in 2019, 610,335 people are living with the virus. This figure varies according on the administrative region, sex, age, and other socio demographic, clinical, and behavioral population factors [2–4].

HIV infection is a varied and heterogeneous epidemic in Ethiopia, with a significant urban concentration and some hotspots driven by high-risk populations, including critical and high-risk group [5–7]. HIV prevalence among Ethiopian female sex workers (FSWs) is estimated to be around 18.5%, which implies that FSWs' sexual partners are significantly exposed to HIV infection and that FSWs may be a major factor in HIV transmission in the community [8]. Ethiopian and other developing country published research have demonstrated that FSWs have a higher likelihood of living with HIV than do general population women in identical reproductive age groups [9–13].

However, It has long been known that using condoms correctly and consistently is an extremely cost-effective global method for preventing HIV infection but Inconsistent condom use (ICU) would pose the greatest proximal to the risk of HIV acquisition and transmission [14]. Despite the fact that the Ethiopian Ministry of Health (MOH) has implemented the strategies and given FSWs priority among MAPP categories for focused intervention, sporadic condom use among FSWs continues to be a significant programming obstacle [5, 7, 10, 15, 16]. The uneven use of condoms by FSWs has been reported in isolated small-scale studies conducted in various locations in Ethiopia, with results ranging from 42% to 52.3% [5, 7, 9].

A number of factors, including aging, exposure to voluntary counseling and testing (VCT), a shorter time spent in commercial sex work, a higher education level, knowledge of HIV infection, nonpaying sexual partnerships, STIs, and alcohol consumption, have been found to be positively and independently, but variably, associated with inconsistent condom use in other studies conducted in Africa, Asia, and Latin America [3, 7, 10, 11, 16–19]. Most of these studies were limited to small geographic areas and did not consider environmental factors which might play significant roles in enhancing inconsistent condom use.

In Ethiopia, FSWs are marginalized by the community since sex selling is considered an illegal income source and a practice against the existing social norms. The prevalence and factors that associated with the inconsistent condom use among FSWs have not been adequately elucidated. Identifying these factors in this population group in regional capitals and major towns will fill gaps. Understanding, the prevalence and associated risk factor about inconsistence condom use among FSWs would inform policy makers to design programmatic interventions in the context of Ethiopia. Therefore, this analysis is conducted to assess the prevalence and factors associated with inconsistent condom among FSWs in Ethiopia.

## Methods

### Study design

This analysis used data from the ‘National HIV and STIs Bio-behavioral Survey (NHSBS)’, which was conducted between December 2019 to May 2020 by using a respondent driven sampling (RDS) technique among FSWs aged 15 years and older who selling sex in selected major cities and towns of Ethiopia. The survey was conducted by the Ethiopian Public Health Institute (EPHI) and Ministry of Health in collaboration with the Federal HIV/AIDS Prevention and Control Office (FHAPCO).

### Study population and setting

The study population was FSWs who used sold sex as source of income in the capital cities in six administrative regions of 10 major towns of Ethiopia. The six cities were selected for the survey includes Addis Ababa (the capital of the Country), Hawassa (Southern Nations Nationalities and Peoples Region/SNNPR), Bahirdar (Amhara Region), Dire Dawa (Dire Dawa City Administration), Harar (Harari Region), and Gambella (Gambella Region) whereas the 10 major selected towns were f Adama, Jimma, Nekemte, and Shashemane (Oromia Region), Arba Minch, Dilla, and Mizan (SNNPR), Gondar and Kombolcha (Amhara Region), and Logia (Afar Region).

### Inclusion criteria

Across the study sites, the participants were FSWs aged 15 years and above who sold sex for at least, four paying partners during the four weeks prior to the survey, who resided or worked at the selected study sites, where they were engaged in selling sex for a minimum of four weeks prior to the survey, and who agreed and provided informed consent to participate in the bio-behavioral survey interview (using a questionnaire) and biological testing, and in possession of a valid coupon.

### Sample size and sampling procedures

The RDS technique was employed to recruit the optimal number of FSWs from sixteen independent study sites. The first study samples (seeds or study triggers) were selected purposively considering the length experience in sex work, and the network study participants had with other FSWs in the selected cities or towns. The seeds were considered as “survey seeds” because they were used as catalysts or bridges to mobilize FSWs through their networks and bringing those who fulfilled the inclusion criteria for recruitment to join their respective peer members form the same city or town. A total of 98 seeds were used to initiate the recruitment of a total of 6,085 FSWs who were included in the study (Table 1).

**Table 1:** The seeds and number of female sex workers included in the survey

Study site	Seeds	Sample size
Adama	8	676
Addis Ababa	13	1101
Arba Minch	5	251
Bahir Dar	8	372
Dilla	5	251
Dire Dawa	5	434
Gambella	6	468
Gonder	5	250
Harar	5	242
Hawasa	8	522
Jimma	5	254
Kombolcha/Dessie	5	251
Logia/Semera	5	251
Mizan	5	255
Nekemte	5	257
Shashemane	5	250
Total	98	6,085

To facilitate the recruitment process, three coupons were issued each of the first seeds to invite three FSWs from among members of her network, which essentially constituted a chain-referral linkage procedure [20, 21]. The coupons remained active starting from the date they were provided to the potential participant and were considered no more active in two weeks, or if the study was completed earlier. The process continued to recruit additional potential participants from the networks until the required sample size was achieved and the RDS equilibrium condition was attained.

### Study variables

The dependent variable under investigation was inconsistent condom usage. If the FSW reported having unprotected intercourse with at least one paying partner within the 30 days before to data collection, it was recorded as "1"; otherwise, it was coded as "0" for consistent condom use. The independent variables which included socio demographic and clinical characteristics (age, marital status, level of education, occupation, average monthly income, STI symptoms, HIV test result, depression), and behavioral and sexual characteristics (age at first sex, age at first sex selling, first sex experience, number of non-paying clients, anal sex practice, number of cities where sex sold, change in location of sex selling, forced sex/rape, alcohol consumption, cigarettes smoking, khat chewing, use of any drugs, u**se** of lubricant during sex, avoidance of condom carrying**,** condom access at workplace, and comprehensive HIV knowledge).

Moreover, six regional cities and ten major towns were included in the analysis to account for heterogeneity across cities and towns, where 16 separate and independent datasets were collected.

Depression severity was measured using the scoring in the Patient Health Questionnaire (PHQ) assessment tool [22] 0–4 “non-minima”, 5–9 “mild”, 10–14 “moderate”, 15–19 “moderately severe” and 20–27 “severe” depression. Alcohol drinking severity level was measured based on the scoring obtained from the Alcohol Use Disorder Identification Test (AUDIT) [23] 0–8 “social drinking”, 9–13 “harmful or hazardous drinking”, and 13–40 “alcohol dependency”. Respondents providing five true answers to the three HIV prevention/treatment and two misunderstanding questions were considered to have “comprehensive knowledge”, and those with less than five correct answers were considered as not having ‘comprehensive knowledge’.

### Data collection and management

After a written informed consent was obtained, each study participant was interviewed in a private location using a pretested standardized bio-behavioral questionnaire by an experienced nurse who received study related training. The questionnaire that was prepared in English was translated into the local language (Amharic) and the data were captured in real time directly on tablets using open data kit (ODK) electronic data management system. The system had a built-in skip patterns and logical validations, and the process was monitored daily to ensure data quality. Sixteen separate cross-sectional data collections were conducted and the RDS assumptions were monitored during data collection by using RDS package that had an inbuilt R statistical software [24]. The survey gathered socio-demographic characteristics, behavior variables, and sexual risk behaviors including inconsistent condom use, and HIV and other STI prevalences.

### Statistical analysis

Statistical analysis was done by using STATA v.16, and R v.3.6.2 statistical software packages. The RDS recruitment process (Tree of recruitment), assessment of the RDS assumptions, and RDS weights generation were done using the RDS package that supported by R statistical software [24]. The RDS assumption check included homophily and convergence of certain main outcome variables of the NHSBS and they were checked for HIV status, inconsistent condom use, and type of FSWs that meet the RDS criteria. The RDS weights were exported by using the RDS-II function to STATA and merged with the whole dataset for further analysis. Descriptive statistics including RDS unweighted and weighted frequencices with proportions, and participants mean age estimate (after checking for normality) were used to summarize the data. Since the data were collected from 16 separate cross sectional study sites, condoms using could be affected by unobserved site-level factors/clusters (cities/towns). Therefore, the models used for analysis have to account for associations among observations within clusters to make efficient and valid inferences. A multi-level logistic regression model was fitted to assess for town and individual level variations simultaneously to adjust hierarchical variations.

In this analysis, a 2-level multilevel mixed-effect logistic regression model was employed. All the independent variables categorized as individual-level variables were considered level-1 variables and the city/town as level-2 variables.Variables with *P*-value ≤ 0.2 in the bivariable analysis were included in the multiple multilevel logistic regression model. In the multilevel regression, the effect of level-2 variable (city/town) was quantified by intra-class correlation (ICC), and the proportion of total variation in the response variable accounted for by the between-city/town variation. The effects of individual-level predictors were quantified by the estimates from the fixed-effect part of the model with a *P*-value less than 0.05 or 95% CI that didn’t include unity.

## Results

### Socio demographic characteristics of participants

Overall, 6,085 FSWs from the six cities and 10 major towns participated in the study. The participants mean (± SD) age was 26.4 (± 6.06) years. Nearly one-half, 2,946 (48%), of them were never married, while about equal number, 2,908 (48%), were divorced, separated or widowed. More than one-half, 3,560 (59%), of them had completed primary school (grades 1–8), and 1,054 (17%) did not attend formal education. Some 5,694 (94%) of the FSWs had their monthly income from sex work, and nearly two-thirds, 2864 (93%) of them had an average monthly income ranging from Ethiopia Birr 5000–7499 (USD 200–300). One in seven (16%) had symptoms of sexually transmitted infection, and 9% of the participants were newly diagnosed with HIV, while 9% of them knew their HIV-positive status before the survey (Table 2). Nearly one-fifth (18%) of the participants had moderate to severe depression.

**Table 2:** Socio-demographic and clinical characteristic of study participants (*N* = 6,085)

Characteristics	Frequency (n)	Percent (%)
**Participant age (years); Mean ± SD (26.4 ± 6.06)**		
15 – 34	5266	86.5
35 – 59	819	13.5
**Marital status**		
Married/Cohabitat	231	4
Divorced/Separated/Widowed	2908	48
Never married	2946	48
**Education status**		
Non-formal education	1054	17
Primary cycle (grade 1–8)	3560	59
Secondary school and above	1471	24
**Occupation**		
Sex work	5694	94
Other^a^	391	6
**Average monthly income (ETB)**		
< 2500	1,778	29
2500 – 4999	2066	34
5000 – 7499	1175	19
7500 +	1066	18
**Sexually transmitted infection symptoms in the last 12 months**		
Yes	1002	16
No	5083	84
**HIV test result**		
Tested new with negative result	4945	81
Tested new with positive result	565	9
Not new tested but positive	575	9
**Depression**		
Not depressed	2468	41
Mild depression	2525	41
Depression	1092	18

^a^‘Others’ category includes FSW with additional income small business (Guilt)

### Behavioral and sexual practice

Behavioral and sexual characteristics of participants are summarized in Table 3. Forty percent (2,430) of them had first sex before age of 15 years, 2,328 (38%) had started selling sex before of 20 years, and the first sexual encounter was forced in 1,347 (22%) of them. Nearly three-quarters, 4,347 (71%), had no ‘non-paying clients’ in the previous 6 months, 4,933 (81%) sold sex in the same city in the previous three years, 426 (7%) ever had anal sex, and 7 71 (13%) were ever raped sex in the past 12 months. Those who had hazardous alcohol drinking habit constituted 1,210 (20%) and were alcohol dependent 2,257 (37%), and those who smoked cigarette, chewed kat, and used any drugs in the last 30 days, 742 (12%), 3, 827 (63%), and 700 (12%), respectively. Most, 5,300 (87%), did not use lubricant during sex in the preceding 6 months, only 595 (10%) could access condom in the workplace, and 4,449 (73%) of the participants did not have sufficient knowledge about HIV infection.

**Table 3:** Behavioral and sexual characteristics of study participants (*N* = 6,085)

Characteristics	Frequency (n)	Percent (%)
**Age at first sex**		
< 15	2430	40
16 – 20	3384	56
> 21	271	4
**Age at first sex selling**		
< 20	2328	38
20– 24	2348	39
> 25	1406	23
**First sex experience**		
Wanted	4738	78
Forced	1347	22
**Number of non-paying clients past 6 months**		
0	4347	71
1	1404	23
2 or more	334	5
**Ever had anal sex**		
Never	5659	93
Yes	426	7
**Number of sex selling cities in the last 3 years**		
Same town	4933	81
1 more town	776	13
2 or more towns	374	6
**Changed location of selling sex in the past 6 months**		
No	4572	75
Yes	1513	25
**First sex experience**		
Wanted	4738	78
Forced	1347	22
**Ever been raped sex in the past 12 months**		
Yes	771	13
No	5314	87
**Alcohol drinking habit (AUDIT)**		
Not Risky	2594	43
Hazardous drinking	1210	20
Alcohol dependence indication	2257	37
**Cigarettes smoking in the last 30 days**		
No	5343	88
Yes	742	12
**Chewing khat in the last 30 days**		
Yes	3827	63
No	2258	37
**Used any drugs**		
Yes	700	12
Never	5385	88
**Used lubricant during sex in the last 6 months**		
Yes used	785	13
Not use	5300	87
**Avoided carrying condom in fear of trouble with police**		
Yes	494	8
No	5591	92
**Access to condom in the workplace**		
Yes	5490	90
No	595	10
**Comprehensive HIV knowledge status**		
Sufficient	1636	27
Not sufficient	4449	73

### Prevalence of inconsistent condom use

**Fig. 1:**
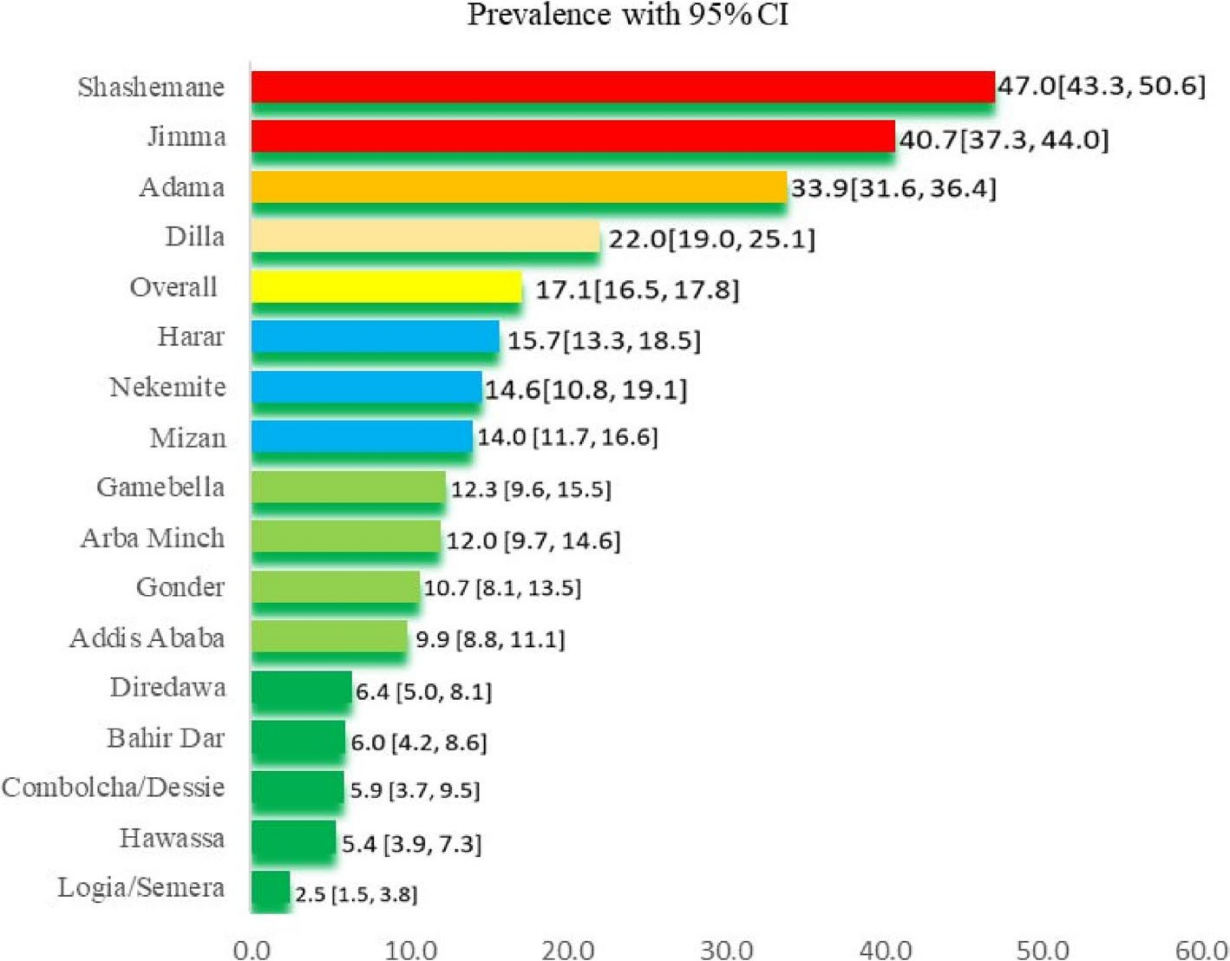
Prevalence of inconsistent condom use in 16 study cities and towns (*N* = 6,085)

The overall, weighted proportion of inconsistent condom use in the cities and towns was 17.1% [95% CI (16.5, 17.8)] (Fig. 1). Of the study sites, Shashemene and Jimma towns had high prevalence rates of inconsistent condom use, 47% and 40.7%, respectively, compared to the others, followed by Adama town with inconsistent condom use prevalence of 34%. The lowest prevalence of inconsistence condom use was reported from logia town (2.5%) and Hawassa city (5.4%).

### Factors associated with inconsistence of condom use

The results of bivariable and multivariable multi-level mixed effect logistic regression analyses are given in Table 4. The results of the multi-level mixed-effect logistic regression model show that the model fitted well for the data over the standard logistic regression to determine the factors that influence the prevalence of inconsistent condom use (${X}^{2}=186.4,$ *P* < 0.01). The result of the intra-class correlation (ICC) of the two-level (multilevel model) revealed that about 11% [95% CI: (0.05, 0.2)] of the variation in the likelihood of inconsistent condom use was explained by the variation among the cities/towns in Ethiopia. The variation between the cities and major towns is statistically significant, as shown by the 95% CI (0.04, 0.2), so any estimation that does not take this effect into account will be biased.

**Table4:** Results of regression analysis of independent variables on inconsistent condom (*N* = 6,085)

Characteristics	Inconsistent condom use		COR (95% CI)	*P*-value	AOR (95% CI)
	No	Yes			
Marital status					
Married/Cohabitating	178	53	1.81 (1.27, 2.57)	0.001	1.56 (1.06, 2.31)^a^
Divorced/Separated/Widowed	2468	440			1
Never married	2473	473	1.20 (1.03, 1.39)	0.021	1.23 (1.04, 1.46)^a^
Education status					
Non-formale education	878	176	1.18 (0.97, 1.44)	0.099	1.32 (1.06, 1.65)^a^
Primary cycle (grade 1–8)	3016	544			1
Secondary school and above	1225	246	1.14 (0.96, 1.36)	0.133	1.15 (0.95, 1.39)
Age category					
15 – 34	4443	823			1
35 – 59	676	143	1.05 (0.85, 1.30)	0.15	1.29 (1.01,1.64)^a^
Depression level					
Not depressed	2234	234			1
Mild depression	2028	497	2.02 (1.69, 2.41)	< 0.0001	1.49 (1.23,1.80)^a^
Moderate to severe depression	857	235	2.28 (1.83, 2.84)	< 0.0001	1.43 (1.13,1.82)^a^
Alcohol drinking (AUDIT)					
Not sisky (social) drinking	2345	249			1
Hazardous drinking	1058	152	1.19 (.95, 1.49)	0.130	1.05 (0.83, 1.33)
Alcohol dependence indication	1702	555	2.42 (2.03, 2.88)	< 0.0001	1.58 (1.29,1.92)^a^
Used of any drug					
No	4616	769			1
Yes	503	197	2.06 (1.68, 2.53)	< 0.0001	1.50 (1.20, 1.88)^a^
Ever been raped to have sex in the past 12 months					
No	4029	709			1
Yes	1090	257	1.33 (1.12, 1.57)	0.001	1.75 (1.43, 2.16)^a^
Selling Sex location change of in the past 6 months					
No	3969	603			1
Yes	1150	363	1.61 (1.37, 1.89)	< 0.0001	1.27 (1.06,1.51)^a^
Number of cities worked sex selling in the last three years					
Same town	4245	688			1
1 more town	613	163	1.54 (1.25, 1.89)	< 0.0001	1.77 (1.48,2.11)^a^
2 or more towns	259	115	1.96 (1.51, 2.56)	< 0.0001	3.01 (2.27,3.99)^a^
Number of non-paying partners in the past 6 months					
Never	3855	492			1
Only one	1074	330	2.14 (1.81, 2.52)	< 0.0001	1.77 (1.48,2.11)^a^
2 and more	190	144	4.63 (3.58, 6.00)	< 0.0001	2.99 (2.26, 3.95)^a^
STI symptoms at least two in the last 12 months					
No	4370	713			1
Yes	749	253	1.96 (1.64, 2.33)	< 0.0001	1.27 (1.04, 1.55)*
HIV comprehensive knowledge					
Not sufficient	3695	754	1.40 (1.17,1.66)	< 0.0001	1.39 (1.15,1.68)^a^
Sufficient	1424	212			1
Avoided caring condom in fear of trouble with police					
No	4807	784			1
Yes	312	182	3.03 (2.44, 3.75)	< 0.0001	1.98 (1.56, 2.52)
Ability to get condom in the sex workplace					
No	412	183	2.08 (1.69, 2.57)	< 0.0001	2.13 (1.69, 2.67)^a^
Yes	4707	783			1
HIV test result					
Tested new negative	4184	761			1
Tested new Positive	436	129	1.47 (1.17, 1.84)	0.001	1.52 (1.19,1.95)^a^
Alread known positive	499	76	1.07 (0.82, 1.40)	0.619	1.02 (0.76, 1.37)
Random Effect: Study Site Var (cons)					0.38 (0.18,0.81)
ICC://Intra class correlation//					0.11(0.05, 0.20)^a^
LR test vs. logistic model				0.01	${X}^{2}=186.4,$

^a^Statistically significant

As shown in Table 4, married or cohabitation status among the FSWs was significantly and independently associated inconsistent condom use compared with the divorced or widowed group [AOR = 1.56; 95% CI (1.06, 2.31)], as for those who were never married [AOR = 1.23; 95% CI (1.04,1.46)]. Similarly, inconsistent condom use was significantly associated with severe depression compared with those with no depression [AOR = 1.49;95% CI (1.18, 1.9)], alcohol dependence compared with non-risky alcohol intake [AOR = 1.56;95% CI (1.06, 2.31)], and any drug use compared with who used none [AOR = 1.43;95% CI (1.14–1.79)]; having non-formal education compared with primary school attendance [AOR = 1.32; 95% CI (1.06, 1.65], being in the age group 35–59 years compared with those 15–34 years [AOR = 1.29; 95% CI (1.01, 1.64)], and having a history of sexual violence during 12 previous month by partners [AOR = 1.75; 95% CI (1.43, 2.16)]. Likewise, inconsistent condom use significantly associated with changed sex work location compared with those who did not [AOR = 1.27;95% CI (1.06, 1.51)], Sex selling in more than two cities or towns during the last three years [AOR = 3.01;95% CI (2.27, 3.99)], practicing anal sex who had none [AOR = 2.74;95% CI (2.15, 3.5)], and having one more and two or more nonpaying sexual partners compared with those who only had one [AOR = 1.77; 95% CI (1.48,2.11)] and [AOR = 2.99; 95% CI (2.26, 3.95)], respectively.

Independent and significant association was documented between inconsistent condom use among those who had STI symptoms, at least, two time per year than those who did not have STI [AOR = 1.27; 95% CI (1.04, 1.55)], and experiencing sexual violence (rape or forced sex) compared with none [AOR = 1.75; 95% CI (1.43, 1.55)]. Inconsistent condom use was also independently associated with and insufficient HIV knowledge of the FSWs [AOR = 1.39; 95% CI (1.15,1.68)], newly testing HIV positive compared to those new testing negative [AOR = 1.52; 95% CI (1.19, 1.95)], not carrying condoms for fear trouble from the police compared with those with no such a fear [AOR = 1.98; 95% CI (1.56, 2.52)], and lack of access to condom at the workplace [AOR = 2.1.3; 95% CI (1.69, 2.67)]. The result of the intra-class correlation (ICC) of the two-level (multilevel model) revealed that about 11% [95% CI: (0.05, 0.20)] of the variation in the likelihood of inconsistent condom use was explained by the variation among the cities/towns in Ethiopia.

## Discussion

The analysis of our data from the NHSBS study shows that the prevalence of inconsistent condom use among FSWs in 2020 was 17.1%, which is lower than the prevalence reported from Ethiopia by isolated small-scale studies conducted in Gondar town of 52.3% [5], Bahirdar town of 55.3% [25], and Dessie town of 38% and 55% [7, 9]. Our finding is also lower than what has been reported by other studies conducted elsewhere in similar settings like Uganda and India, which have shown that the prevalence of inconsistent of condom use is in the order of 40% and 37.5% [16, 18], respectively. The variation in prevalence of inconstant condom use among the small-scale isolated study reports as well as their difference from our finding are expected because our analysis was based on a large sample of FSWs working in multiple regional and sub-regional cities and towns. The variations could also be because of the differences in geographic setting, study design, and study period. Nonetheless, a substantial work still needs to be done to further explain this variations in condom use among the most at risk groups, FSWs in particular, as emphasized by the evidence from a systematic review [2].

The odds of being inconsistent condom use was significantly higher among those who were married/cohabiting, had moderate to severe depression, were alcohol dependent, used any drug, were in the older age group, had a history of sexual violence, had sex selling location changes, had length of sex selling experience, had anal sex experience, had a number of non-paying sexual partners, had sex selling experience in more than two cities, had experience of rape or forced sex, were newly HIV diagnosed, who lacked of condom access at the workplace, and did not have comprehensive HIV knowledge.

The older age group (35–59 years) who were more experienced in sex selling was more 1.29 times more likely to use condom inconsistently than their younger counterparts. This could possibly be because the older age group were more experienced in the business for a longer period of time and were not open to changing their practice. Indeed, this observation is consistent with previous reports from Ethiopia, Ghana, Pakistan, and India, which showed that inconsistent condom use, was more likely among older FSWs [10, 16, 26]. An earlier study among younger FSWs also showed that this group used condoms more consistently than older FSWs because the younger group might have the power to negotiate condom use and refuse unsafe sex due to they may had more opportunity to be being preferred from older groups [19].

FSWs with married or cohabiting and never married were more likely for inconsistent condom use than the divorced/separated/widowed group. That may be they might not want to use condoms with their husbands or boyfriends during sex practice by bearing in mind as they might need to have children, building trust with their partner, and perceive condom use would reduce sexual pleasure. Other studies conducted both in Ethiopia [7] and elsewhere in Africa [27] have reported similar findings, but a report from Uganda showed that currently married women were less likely to report inconsistent condom use with paying clients the possible variation could be due to differences in study design and sample size [18].

Our finding revealed that FSWs who were with no formal education were 1.32 times more likely to be at risk for inconsistence condom use compare with counter parts or as of the educated group that might be the uneducated group might have less confidence to use condoms with their sexual partners due to the lack of comprehensive knowledge about condom use and its contribution in preventing HIV or other STIs transmission. This is also supported by the findings of studies conducted in Ethiopia and Laos [5, 28], which showed that educated FSWs had, unlike their non-educated counter parts, the ability to negotiate condom use with their sexual partners.

Our analysis identified that FSWs with alcohol dependence and sexual violence experience were 1.58 and 1.75 times more likely to have inconsistence condom use with either regular or occasional sex partners than their counterparts, respectively. These drivers were independently associated with inconsistent condom use. Alcohol consumption is common among FSWs and their sexual partners before having sex and this, which might change their behavior and encourage them to have sex without condom, predisposing them to new HIV infection and other STIs. Evidence from previous similar studies conducted in Ethiopia, Zambia, South Africa, and Pakistan has also shown that FSWs who were ever used alcohol had a lower likelihood of consistent condom use than women who never used alcohol [16, 29, 30]. Similarly, in our analysis sexual violence compromises FSWs’ negotiation ability and decision-making capacity about condom use with their sexual partners, and this finding is consistent with reports from previous studies in Laos and Pakistan emphasizing that FSWs were likely to be physically abused and forced to have sex without condoms [16, 28, 31].

Our finding also showed that FSWs who lacked access for condom in the workplace were two times more likely to use condoms inconsistently than those who did not have access. This indeed is now a common observation as also shown by findings of different studies conducted in other similar settings like Pakistan, the Democratic Republic of Congo, Zambia, and Ghana [9, 11, 26, 29]. Some independent variables could be improved over time such as access to condom in work place, health education and legal framework to protect female reproductive right and designing of income generation polices and strategies could reduce contribution of negative impact for inconsistence of condom use.

## Limitation

As part of the ongoing surveillance, the survey had targeted only provincial (regional) capital cities and major towns. Even in the selected cities and town, the presence of harder-to-reach sex worker groups like home-based sex workers might not be fully accounted for. Lack of detailed city maps for all regional capital cities and major town, including street names, was a challenge for mapping and presenting the results in this size estimation study. As this was a cross-sectional study, temporal relationships between inconsistent condom use and the associated factors couldn’t be established.

## Conclusion

The inconsistent usage of condoms among FSWs went against the notion that condom is crucial for preventing the spread of STIs like HIV. Explanatory factors independently associated with inconsistent condom use among FSWs included married/cohabitant status, low educational level, older age, presence of moderate/severe depression, alcohol dependence, any drug use, lack of HIV knowledge, sexual violence, number of sex selling cities, number of nonpaying partners, new HIV diagnosis, and lack of access to condoms in work place, health policy makers and managers, related programs in other relevant sectors like the Ministry of Women and Social Affairs of Ethiopia, and healthcare professionals should take these factors into account in their efforts to improve consistent condom use among FSWs. Similar epidemiological studies are also required to measure the magnitude of the problem among other most at risk populations.

## Data Availability

The datasets used and/or analyzed during the current study available from the corresponding author on reasonable request.

## Abbreviations

AOR: Adjusted Odds Ratio
AUDIT: Alcohol Use Disorder Identification Test
CI: Confidence Interval
COR: Crude Odds Ratio
EPHI: Ethiopian Public Health Institute
FHAPCO: Federal HIV/AIDS Prevention and Control Office
FSWs: Female sexual workers
ICC: Intra-Class Correlation
MARP: Most at Risk Population
MOH: Ethiopian Ministry of Health
NHSBS: National HIV and STIs Bio-behavioral Survey
ODK: Open Data Kit
PHQ: Patient Health Questionnaire
PLHIV: People Living with HIV
RDS: Respondent Driven Sampling
SD: Standard Deviation
TB: Tuberculosis
SNNPR: Southern Nations Nationalities and Peoples Region
SSA: Sub-Saharan Africa
STIs: Sexually Transmitted Infections
VCT: Voluntary Counseling and Testing
